# Integration of QTL, Transcriptome and Polymorphism Studies Reveals Candidate Genes for Water Stress Response in Tomato

**DOI:** 10.3390/genes11080900

**Published:** 2020-08-07

**Authors:** Isidore Diouf, Elise Albert, Renaud Duboscq, Sylvain Santoni, Frédérique Bitton, Justine Gricourt, Mathilde Causse

**Affiliations:** 1 INRAE, UR1052, Génétique et Amélioration des Fruits et Légumes, 67 Allée des Chênes, Centre de Recherche PACA, Domaine Saint Maurice, CS60094, 84143 Montfavet, France; dioufisi@hotmail.com (I.D.); alberte2@msu.edu (E.A.); renaud.duboscq@inrae.fr (R.D.); frederique.bitton@inrae.fr (F.B.); justine.gricourt@inrae.fr (J.G.); 2 INRAE, UMR1334, Amélioration génétique et Adaptation des Plantes, Montpellier SupAgro-INRA-IRD-UMII, 2 Place Pierre Viala, 34060 Montpellier, France; sylvain.santoni@inrae.fr

**Keywords:** RNA sequencing, water deficit, transcriptome, genotype × watering regime interaction

## Abstract

Water deficit (WD) leads to significant phenotypic changes in crops resulting from complex stress regulation mechanisms involving responses at the physiological, biochemical and molecular levels. Tomato growth and fruit quality have been shown to be significantly affected by WD stress. Understanding the molecular mechanism underlying response to WD is crucial to develop tomato cultivars with relatively high performance under low watering conditions. Transcriptome response to WD was investigated through the RNA sequencing of fruit and leaves in eight accessions grown under two irrigation conditions, in order to get insight into the complex genetic regulation of WD response in tomato. Significant differences in genotype WD response were first observed at the phenotypic level for fruit composition and plant development traits. At the transcriptome level, a total of 14,065 differentially expressed genes (DEGs) in response to WD were detected, among which 7393 (53%) and 11,059 (79%) were genotype- and organ-specific, respectively. Water deficit induced transcriptome variations much stronger in leaves than in fruit. A significant effect of the genetic background on expression variation was observed compared to the WD effect, along with the presence of a set of genes showing a significant genotype × watering regime interaction. Integrating the DEGs with previously identified WD response quantitative trait loci (QTLs) mapped in a multi-parental population derived from the crossing of the eight genotypes narrowed the candidate gene lists to within the confidence intervals surrounding the QTLs. The results present valuable resources for further study to decipher the genetic determinants of tomato response to WD.

## 1. Introduction

Drought is among the most common abiotic stress factor affecting plant growth and crop yield, and more frequent episodes of drought are expected to arise with climate change [1]. Extensive research has been dedicated to understanding the mechanisms driving plant adaptation to drought [2]. Water deficit (WD) stress induced by drought can be defined as a period of plant exposure to dry soil, subsequently resulting in reduced growth and yield [3]. A global understanding of the complex interplay between genetic and environmental factors in crop adaptation to WD is therefore a key aim for breeding purposes.

The phenotypic changes triggered by WD—encompassing yield decrease—are inherent to the process of plant acclimation through physiological and molecular regulation. Indeed, WD disrupts cellular homeostasis, eliciting signaling cascades and the regulation of several physiological processes, notably osmotic adjustments—through the accumulation of compatible solutes—activation of the antioxidant defense system and variation in plant hormone concentrations [4]. Mild to severe WD stress is generally associated with changes in gene expression and the regulation of different stress-responsive genes. Hundreds of genes showing susceptibility to WD stress have been identified in the model plant *Arabidopsis thaliana* [5] and in major crops such as tomato [6], wheat [7], maize [8] and rice [9].

Crops usually show high sensitivity to WD, especially when it occurs during the reproductive stage [10]. However, the degree of sensitivity to WD can vary widely between cultivars/genotypes within a species. Genetic determinants of plant responses to WD have been studied in several species and genotype–phenotype associations under WD conditions have yielded many quantitative trait loci (QTLs) affecting plant responses to WD [11,12,13]. Tomato growth and development are affected by WD stress and an extensive genotype × watering regime interaction (G × W) has been observed in different experimental populations for the crop [14,15,16,17,18]. Water deficit-responsive QTLs have been identified using agronomic traits, as well as eco-physiological modeling parameters [14,18,19]. However, the genomic regions covered by the QTLs usually cover many genes, limiting our ability to identify the causal genes. High-throughput sequencing technologies give the opportunity to bridge this gap through the analysis of gene expression.

A significant number of gene expression studies have been reported during recent decades, highlighting the effect of gene expression level on phenotypic variation [20,21,22]. Besides, the changes in the gene expression level could vary under different environmental conditions, as well as according to the genetic background [23,24,25]. A promising and reliable approach to identify stress tolerance genes and elucidate the molecular mechanisms and biological pathways involved in abiotic stress adaptation lie, therefore, in the analysis of transcriptome variation at both genotype and environmental condition levels. Few studies have depicted the transcriptome variation under WD in tomato and most of them included only one or two genotypes, usually characterized as WD-tolerant/susceptible [26,27,28]. A recent study, however, characterized differentially expressed genes (DEGs) under WD in large and small tomato fruit accessions and their F1 hybrids, highlighting the presence of the G × W interaction at the gene expression level, and identified interactive expression QTLs [6].

In the present study, we aimed to assess the impact of watering regime condition (W) and G × W interaction on the transcriptome variation of eight diverse tomato genotypes. These eight genotypes have been previously characterized at the phenotypic level for agronomic and physiological responses to WD, highlighting genotype-dependent responses and suggesting genotype-specific adaptive strategies [18,29]. Furthermore, the eight genotypes constituted the parental lines of a multi-parent advance generation intercross (MAGIC) population that was first described in Pascual et al. (2015) and used in Diouf et al. (2018) to identify WD response QTLs [18,30]. Through differential expression analysis, we identified several genes significantly impacted by WD in leaves and fruit pericarps. Genes showing different expression between the control and WD conditions and a significant G × W interaction were highlighted and examined for their co-location with previously identified WD QTLs [18].

## 2. Materials and Methods

### 2.1. Plant Materials 

Eight tomato lines used to generate the MAGIC population presented in Pascual et al. (2015) [30] were used in this study. The eight genotypes belong to different genetic groups, with four genotypes, Cervil (Cer), Criollo (Crio), PlovdivXIVa (Plov) and LA1420 (LA14), from the *Solanum lycopersicum cerasiforme* group (SLC) and the four others, Levovil (Lev), Stupicke Polni Rane (Stup), Ferum (Fer) and LA0147 (LA01), from the *Solanum lycopersicum lycopersicum* group (SLL). These genotypes were selected from a collection of 360 accessions [31] to uncover the maximum genetic diversity of this panel. The experimental design and plant growth conditions are described in detail in Albert et al. (2018) [6]. Briefly, the eight genotypes were grown in spring–summer 2015 in a greenhouse in Avignon (south of France) under two watering conditions: control and water deficit (WD). The control condition consisted of full irrigation treatment according to evapotranspiration (ETP), while the WD condition was set progressively after the flowering of the second truss of Cervil (earliest genotype): the water supply was reduced by 25% compared to the control for one week, then decreased by 40% until the end of the experiment to apply a mild water deficit. Throughout the experiment, the relative humidity of the peat substrate was controlled with a GRODAN^®^ moisture probe and monitored in drought pots at around 30%. Three plants per watering regime and accession were grown in the greenhouse, in four-liter (L) plastic pots filled with peat (Klasmann 165) and watered with nutritive solution (2, 4, 6 mmol L^−1^ of N, P and K, respectively). Temperature, light and air humidity were scored every hour. On average, the temperature was 25 °C during the day and 18 °C during the night, the daily light integral ranged from 5 to 11 MJ m^−2^ day^−1^ and the average humidity was 47% during the daytime and 70% at night. Phenotypic measurements were carried out for different traits related to plant development and fruit composition. Plant height (distance from the soil to the 4th truss) and stem diameter (diameter just below the 4th truss) were measured, with one measure recorded for each plant. Flowering time (flw) was measured as the number of days after sowing date. For each genotype, ten fruits were harvested and weighed to measure the average fruit weight (FW) from the 3rd to the 6th truss. Fruits were then pooled in three biological replicates to measure fruit composition traits, from which dry matter weight (DMW) was evaluated by drying fruit pericarps in an oven at 60 °C for four days. Half of each fruit pool was blended to measure pH and soluble solid content (SSC). Fresh pericarps were also sampled from each pool and frozen in liquid nitrogen before being ground into powder for sugar (glucose and fructose, g 100 g 1 FM) and total vitamin C (VitC, mg 100 g 1 FM) measurements, following the protocols used in Albert et al. [6].

### 2.2. Statistical Analyses of Phenotypic Data

To test for a WD effect at the phenotypic level, a two-way analysis of variance (ANOVA) was performed for each trait separately. The level and significance of the G × W interaction was assessed with the following model: $y_{ij}=\mu +G_{i}+W_{j}+GxW_{ij}+\varepsilon_{ij}.$ In this model, $y_{ij}$ represents the phenotype of genotype i ($G_{i}$) in watering condition j ($W_{j}$) and $GxW_{ij}$ and $\varepsilon_{ij}$ are the genotype × watering regime interaction and residual errors, respectively. For each trait, phenotypic plasticity (PP) was calculated according to the following formula: $PP_{i}=(y_{WD}-y_{control})/y_{control}\;$, where $PP_{i}$ represents the plasticity (WD response) for each genotype (i) and $y_{WD}\;\left(\mathrm{or}\;y_{control}\right)$ the average phenotype in the WD (or control) condition. The average response of the eight genotypes was further evaluated by computing the relative stress impact: $RSI=\left({\bar{Y}}_{WD}-{\bar{Y}}_{control}\right)/{\bar{Y}}_{control}$, where $\bar{Y}\;$represents the average value across all genotypes for a trait, given a condition.

### 2.3. RNA Extraction

For each genotype, total RNA was collected from growing leaves and fruit pericarps (at least five fruits) at the cell expansion stage. Given the differences in their phenological stage, the cherry (SLC) and large fruit accessions (SLL) were sampled for fruit pericarps at 14 and 21 days after anthesis (DAA), respectively. The samples were immediately frozen after collection, then pooled per genotype, organ and condition, with two to three biological replicates. Messenger RNA (mRNA) was extracted using the Spectrum Plant Total RNA Kit and assessed on a Nanodrop 1000. A total of 72 paired-end strand-specific libraries were generated from 1 µg of the total RNA and sequencing was performed on a Hiseq 3000 with the GenoTool platform (INRA Toulouse). The biological replicates in both conditions were disposed in the same lane for each genotype. Detailed information about the RNA extraction protocol and read sequence processing are described in Albert et al. (2018) [6].

### 2.4. Differential Gene Expression Analysis

Differential expression (DE) analysis was performed using a negative binomial generalized linear model with the Bioconductor and R package *DESeq2 1.14.1* [32]. The impact of WD on transcriptome variation was evaluated in fruit and leaf samples separately. The analysis was restricted to genes with at least 20 read counts across samples, encompassing 23,552 and 22,864 genes for leaf and fruit samples, respectively. The impact of genotype and watering condition on transcriptome variation was first graphically evaluated through principal component analysis (PCA) on the normalized gene expression. Normalized counts were transformed with the variance stabilizing transformation (VST) before performing the PCA analysis. Thereafter, genes showing differential expression according to the watering condition were identified for each genotype and for fruit and leaf organs separately. A False Discovery Rate (FDR) threshold of 5% [33] was applied to call significantly differentially expressed genes using the Wald test.

### 2.5. Two-Way ANOVA of Transcript-Level Variation

Analysis of variance was performed on the expression level using the expressed genes (same gene set as the DE analysis) to measure, for each single gene, the relative contribution of genotype (G), watering condition (W) and G × W factors to gene expression variation. The ANOVA model used was similar to the one described for phenotypic trait analysis, with the only difference being that $y_{ij}$ this time represents the transcript abundance (VST-transformed values of normalized counts) of genotype i ($G_{i}$) in watering condition j ($W_{j}$) for each single gene included in the analysis. A FDR threshold of 5% was applied to call significant differences [33]. This analysis was conducted for each organ separately and the proportion of the sum of squares attributed to genotype (G), watering condition (W) and G × W factors were retrieved and used to estimate the relative contribution of each factor.

### 2.6. Gene Ontology Enrichment Analysis

Gene ontology (GO) enrichment analysis was performed using the R–Bioconductor package *goseq* (version 1.36.0) [34]. Enriched GO terms were investigated for the sets of DEGs in fruit and leaves separately. For each organ, the DEGs were separated into three different categories (up, down and up-down) depending on the pattern of the expression regulation. The up (or down) category involves all DEGs that were consistently up- (or down-) regulated across genotypes or in at least one genotype. The up-down category involved DEGs showing different regulation patterns (i.e., upregulated in one genotype and downregulated in another and vice-versa). Only annotated genes were considered for the GO analysis. Significant GO terms for biological processes (BPs) and molecular function (MF) were selected after multiple testing correction by setting an FDR threshold at a 5% cutoff with the Benjamin and Hochberg method [33]. The SL2.50 version of the reference genome “Heinz” was used and correction for length bias was carried out with the *nullp* function before GO enrichment testing. The gene space was composed of 27,014 genes, among which 18,837 genes presented at least one piece of GO information. 

### 2.7. Co-Localization of the DEGs and Tomato WD QTLs 

Diouf et al. (2018) conducted a QTL mapping analysis using a MAGIC population generated from the intercross of the eight genotypes described in the present study under control conditions and WD [18]. The WD stress condition consisted of a 40% reduction in water irrigation. The authors identified 12 WD-responsive QTLs, among which nine were specific to the WD condition and three QTLs were detected using phenotypic plasticity in response to WD for different traits related to plant and fruit growth. The respective genomic regions of these QTLs were compared to the locations of DEGs identified across the eight parental lines. The comparison was made possible using the physical positions of the tomato reference genome (version SL2.50). Besides, the correlations between the expression level and phenotypic allelic effect for each QTL were assessed.

## 3. Results

### 3.1. Phenotypic Response to WD

**Figure 1:**
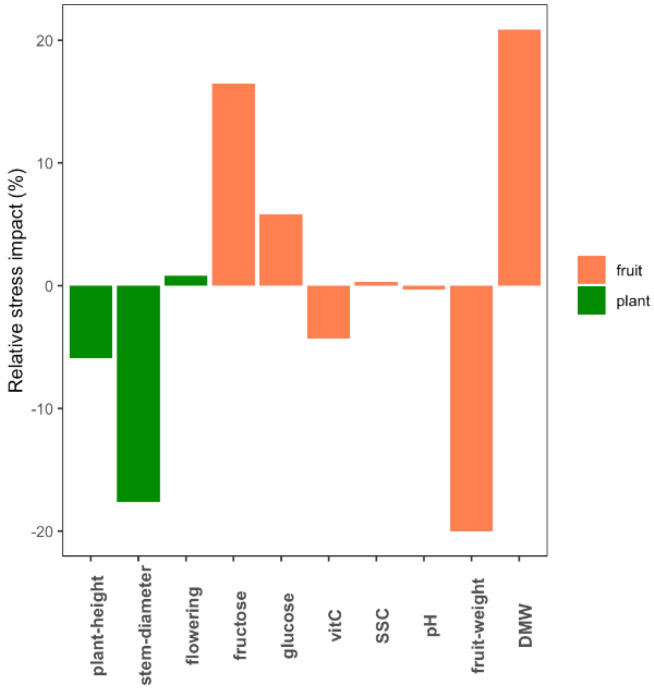
Average impact of water deficit (WD) at the phenotypic level across the eight genotypes. The bar plots indicate for each trait the proportion by which WD decreased/increased the average value of the eight genotypes.

A total of 10 phenotypic traits related to plant development and fruit composition were measured in eight tomato genotypes grown under control and WD conditions. The genotypes significantly differed for all the phenotypic traits measured (*p*-value < 0.05) and WD treatment significantly affected every trait except flowering time, glucose, pH and SSC. Considering the average response across genotypes, fruit dry matter weight (DMW +21.4%) and fruit fresh weight (FW −20.7%) were the most affected traits (Figure 1). Variability was observed among the genotypes in their response to WD at the phenotype level (Table S1). Ferum, for instance, showed the highest susceptibility to WD compared to the other genotypes for most of the traits (Figure S1). The G × W interaction was only significant for four traits (height, diameter, FW and glucose).

### 3.2. Transcriptome Variability Across the Eight Genotypes

**Figure 2:**
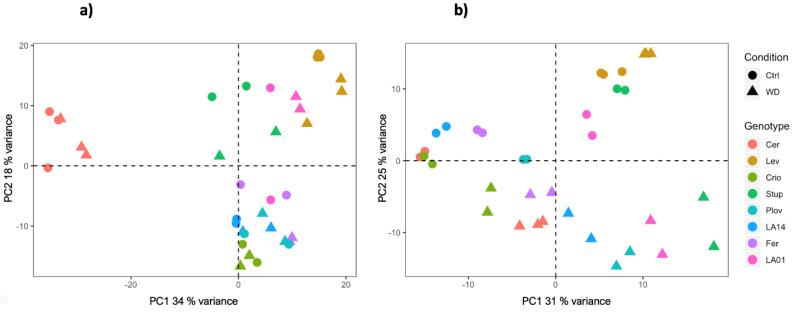
PCA plot of the normalized read counts in fruit (**a**) and leaf (**b**) samples, grown in control or WD conditions.

RNA sequencing was carried out using growing leaf and fruit pericarp samples at the cell expansion stage, collected from the eight genotypes under control and WD conditions. Overall, the RNA sequencing process yielded a total of 23,552 genes (67.8% of tomato CDS) and 22,864 genes (65.8% of tomato CDS) with expression levels above background noise, in leaves and fruit, respectively. Principal component analysis (PCA) on the transformed normalized read counts showed a clear clustering of the samples according to genotypes and conditions (Figure 2). The first two axes of the PCA explained about 52 and 56% of the variation of the gene expression level in fruit and leaf samples, respectively. For both organs, variability in the transcript levels according to genotypes was captured by both the PC1 and PC2 axes. In addition, for leaf samples, the PCA plot separated the conditions following the PC2 axis, Cervil and Levovil appeared as the most discriminant genotypes and, for fruit organs, transcriptomic variation was highly specific for Cervil, as observed in the PCA plot (Figure 2).

### 3.3. DEGs Under WD Conditions

**Figure 3:**
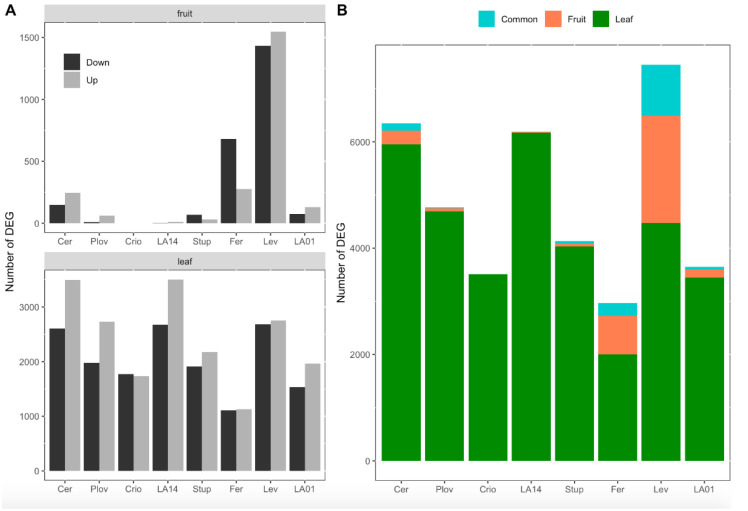
Number of differentially expressed genes (DEGs) per genotype and organ. (**A**) Number of down- and upregulated genes in response to water deficit in fruit (top) and leaves (down). (**B**) Proportion of genes that were significantly differentially expressed in response to water deficit in leaves only (green), in fruit only (red) or in both organs (blue). The eight genotypes were ordered according to their genetic group, the first four genotypes being cherry accessions (SLC) and the last four, large fruit accessions (SLL).

Considering the eight genotypes together, a total of 4132 and 12,938 DEGs between the two watering conditions were identified in the DE analysis in fruit pericarps and growing leaves, respectively (Tables S2 and S3). The number of DEGs was variable among genotypes. In fruit, the number of DEGs varied from zero (Criollo) to 2978 (Levovil) and, on average, the SLL accessions showed a higher number of DEGs than the SLC genotypes (Figure 3A). In particular, Levovil and Ferum, two SLL genotypes, presented the highest number of DEGs between the conditions, although the proportion of up/downregulated genes differed. For leaf samples, the number of DEGs was higher for the different genotypes than in fruit. The total number of DEGs in leaves varied from 2240 (Ferum) to 6177 (LA1420) and the proportion of up/downregulated genes was almost balanced except for Cervil and LA1420. Most of the DEGs identified were organ-specific, highlighting the organ-dependent regulation of gene expression under WD (Figure 3B). Indeed, depending on the genotypes, 45 to 82% of the DEGs in fruit were not differentially expressed in leaves.

A small set of genes were been identified as DEGs in both organs (Figure 3B), representing 0–13% of the total DEGs according to the genotype. For most of the genotypes, the pattern of gene expression regulation was different between leaves and fruit. For instance, for genotypes with more than 100 consistent DEGs between fruit and leaves—notably Cervil, Levovil and Ferum—the proportion of the genes upregulated in one organ (leaf or fruit) and downregulated in the other was non-negligible, representing 47, 44 and 79%, respectively (Figure S2).

### 3.4. Genotype and WD Impact on the Transcript Level

A two-way analysis of variance (ANOVA) was performed using the VST-transformed values of the normalized transcript level to assess the effect of genotype, watering condition and G × W interaction on the regulation of gene expression. This analysis revealed a total of 16,392 and 19,450 genes that were affected by at least one of the abovementioned factors in fruit and leaves, respectively. A high proportion of the genes tested showed a significant genotype effect, highlighting an important effect of the genetic background on gene expression regulation among the eight lines (Figure S3). A much smaller number of genes were specifically affected by WD or the G × W interaction.

### 3.5. Gene Ontology Enrichment Analysis

GO enrichment analysis was conducted on a set of 3794 (92% of the total fruit DEGs) and 11,804 (91% of the total leaf DEGs) DEGs in fruit and leaves, respectively, yielding a total of 24 significantly enriched GO terms (Table 1). Only genes with a known annotated function were selected for the GO analysis. GO terms associated with “cell redox homeostasis”, “metabolic process”, “microtubule-based movement” and “protein phosphorylation” were significantly over-represented among the DEGs in leaves. With reference to the molecular function, three GO terms (GO:0016168, GO:0003735 and GO:0008152) related to “chlorophyll binding”, “structural constituent of ribosome” and “metabolic process”, respectively, were enriched considering DEGs in fruit and leaves separately.

### 3.6. DEG Co-Location with Previously Identified WD-Responsive QTLs

**Figure 4:**
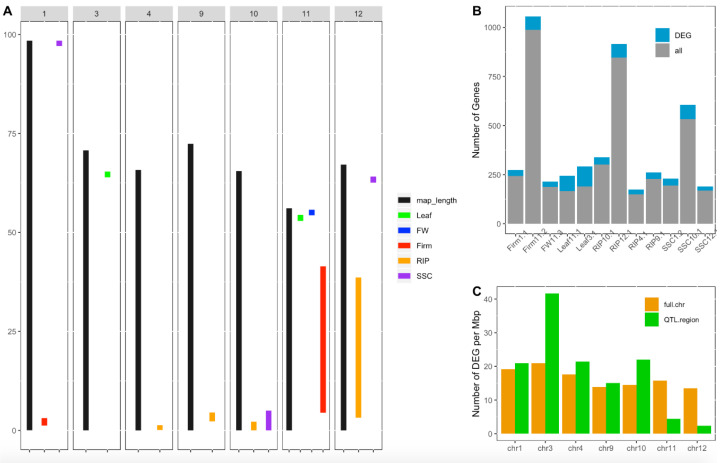
Candidate gene screening for tomato plasticity quantitative trait loci (QTLs). (**A**) Position in Mbp of WD-responsive QTLs identified in the multi-allelic MAGIC population in Diouf et al. (2018) [18]. Black bars represent the chromosome length and colored bars represent confidence interval regions of the plasticity QTLs for different fruit traits assessed: fruit weight (FW), fruit firmness (Firm), fruit ripening (RIP), soluble solid content (SSC), leaf length (Leaf) and flowering time (flw). (**B**) Number of genes within the whole CI region of the QTL (in gray) and number of genes showing significant differential expression under water deficit (in blue). (**C**) Number of DEGs per Mbp within the whole chromosome (in orange) and within the regions covered by QTLs per chromosome (in green).

The combination of gene expression and QTL information was used to identify candidate genes under the WD-responsive QTLs detected in the MAGIC population. To illustrate the approach, we focused on three and nine QTLs previously identified in the MAGIC population using the plasticity response under WD or QTLs identified specifically under the WD condition [18]. The selected QTLs were mapped to seven out of the 12 tomato chromosomes (Figure 4A). The confidence interval regions of these QTLs encompassed 11 to 51 cM and included in their intervals 149 to 988 genes. However, the number of DEGs within the QTL regions varied from 20 to 102 genes, reducing the set of potential candidates by 46–93% according to the QTL (Figure 4B). The number of DEGs per Mbp was assessed for each of the seven chromosomes carrying the WD-responsive QTLs and these genomic regions covered by the WD-responsive QTLs were enriched with DEGs for some chromosomes (Figure 4C). For reasons of consistency, QTLs identified on leaf and fruit traits were mapped specifically to the DEGs detected in leaf and fruit organs, respectively.

**Figure 5:**
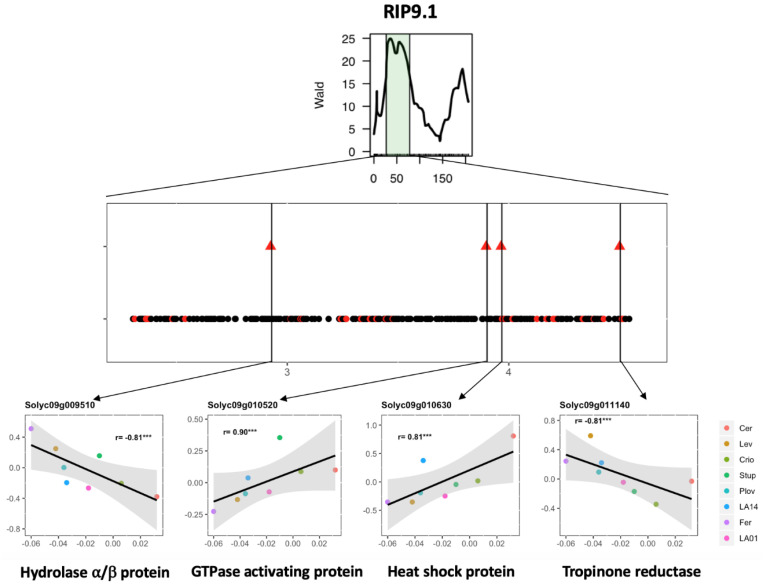
Candidate gene selection for the WD-responsive fruit ripening QTL (RIP9.1) detected in Diouf et al. (2018) [18]. (**Top**) Representation of the RIP9.1 region on chromosome 9 detected using the plasticity of fruit ripening through interval mapping analysis in the MAGIC population. (**Middle**) Genes within the RIP9.1 QTL interval. Black dots represent non-DEGs, and red dots, DEGs in the present study. Triangles represent the DEGs for which the delta expression level (expression level in WD–expression level in control) was significantly correlated to the allelic effect of the QTL for the eight genotypes. (**Bottom**) Correlation between the estimated allelic effect at the QTL (*x*-axis) and the delta log2 expression levels (*y*-axis) for four candidate genes with their functional annotation.

QTL detection using the parental haplotype probabilities in the MAGIC population allows for the estimation of the allelic effect for each parental line at every QTL position. Correlation analysis was further investigated between the allelic effect and expression level across the eight genotypes. The expression of a total of 46 genes showed significant correlation with the QTL allelic effect, reinforcing their potential implication in regulating fruit phenotype variation under WD (Table S4). The whole process of candidate gene selection is depicted in Figure 5 for the QTL RIP9.1 as an example.

## 4. Discussion

Plant response to drought is a complex mechanism which ultimately results in phenotypic changes that can alter agronomic performance in crops. Such responses strongly depend on the genetic background, leading to the necessity of screening different genotypes/accessions for drought tolerance. Genotype specificity has been depicted in tomato response to WD for different agronomic traits [35,36,37]. The present study highlighted a strong genotype-dependent variation under WD at both the phenotype and transcriptome levels.

The WD treatment induced a significant variability in the transcriptome across the eight genotypes. A total of 14,065 DEGs under WD were detected, among which 7393 (53%) were genotype specific. Cervil and Levovil presented the most divergent pattern of gene expression variation at the fruit level (Figure 2), which was consistent with the phenotypic variation since these two genotypes also presented the smallest and largest fruit weight, representing on average 6.1 g and 119 g, respectively. Furthermore, these two genotypes were also identified as highly divergent at the whole genome level when comparing their polymorphism sequences against the reference genome [38]. Moreover, a bi-parental population generated from these two lines yielded the significant discovery of genetic loci controlling trait variation in tomato [6,39,40].

Organ and tissue sampling can significantly alter the gene expression profile in plants [41,42]. Besides, the organ-specific transcript level might be exacerbated by the presence of stress factors, especially at a specific growth stage. The WD treatment in our study was applied from the first inflorescence appearance until the end of the growing season. Fruit sampling was elaborated to limit differences that might arise from growth stages between the SLL (sampled at 21 DAA) and SLC (sampled at 14 DAA) accessions. The low number of fruit DEGs in SLC accessions could have been linked to the sampling strategy we adopted. However, this hypothesis is not supported by the low number of fruit DEGs observed for LA0147 and Stupicke, which are both SLL accessions.

The process of plant adaptation to drought usually starts with cellular sensing and signaling, which activates downstream drought-responsive genes [4]. The activation of the signaling pathway under WD at the early vegetative growth stage is hence expected to lead to better adaptation. This may explain the high number of DEGs in growing leaves. Ripoll et al. (2016) have shown, in the same eight lines, a higher WD impact on leaves than at the fruit level and outlined a prevalence of osmotic adjustment and photosynthetic adaptation in tomato response to WD [29]. The source–sink relationship is highly altered under the WD stress condition in tomato [43], which could eventually reflect different transcriptome responses.

Comparative analysis of the whole transcriptome variation under WD has been conducted in several species; however, most of the time, only two genotypes are included (usually tolerant vs. sensitive genotypes). These classical designs are very powerful to detect DEGs involved in specific stress responses. Yet, the few studies which assessed the impact of a specific stress and the genetic background simultaneously revealed the significant effect of the genotypes with a frequent GxE interaction at the transcriptome level [6,41]. The eight MAGIC parental lines were selected from a panel of 360 tomato accessions to represent the diversity observed within the cultivated tomato. Our results provide evidence of a strong genotype effect at the transcriptome level, independent of the growing condition. Expression QTL analysis in the MAGIC population should then yield significant results that could help to get more insight into the molecular mechanisms shaping tomato variation.

Integrating QTL information and gene expression variation is a common strategy for candidate gene screening [44]. This strategy yielded significant results in MAGIC populations in maize [45] and cotton [46], where the parental haplotypes were exploited to drastically narrow the potential candidate genes. Here, we propose a strategy combining DEGs and WD-responsive QTLs to identify candidate genes affecting WD stress response in tomato. The whole approach depicted in Figure 5 highlighted 46 candidate genes (CGs), which showed expression variability in the eight parental MAGIC lines under the WD condition for 12 WD-responsive QTLs. Four interesting CGs were indeed identified in the region of the RIP9.1 QTL, among which was Solyc09g010630, a gene coding for a heat-shock protein (Hsp). In the literature, Hsp has been described as playing a role in drought tolerance [47,48,49] and fruit ripening in tomato [50]. Regarding the allelic effect of the RIP9.1 QTL, WD stress accelerated ripening in all parents except Cervil and Criollo (Figure 5). A total of 82 polymorphisms (75 SNPs and seven INDELs) were identified for Solyc09g010630, among which 54 discriminated the most divergent lines (Cervil and Ferum) regarding the estimated allelic effect of the QTL. The allelic variants of these polymorphisms are in accordance with the pattern of the gene expression and allelic effects across the eight parental lines. Further studies are, however, necessary to test the potential implication of these alleles in tomato fruit response to WD.

Similarly, other interesting candidates were identified. For instance, RIP10.1 is another QTL detected for the plasticity of fruit ripening under the WD condition, which carried a total of 301 genes within its confidence interval region, among which only 37 genes (12%) showed significant differential expression levels in fruit pericarps. Some of these genes presented a significant correlation between their expression level in response to WD and the allelic effect of the QTL across the eight parental lines, encompassing Solyc10g006130, annotated as “ethylene responsive transcription factor (ERF) 3a” and Solyc10g006650, encoding a “flavoprotein wrbA”. In tomato, ERFs have been described as being involved in the fruit maturation process and also as drought inducible transcription factors [51,52]. Thus, Solyc10g006130 represents an interesting candidate for studying the interaction between drought and fruit maturation in tomato. Solyc10g006650 also constitutes a good candidate, as the role of flavoprotein in tolerance to osmotic stress has been depicted in *Arabidopsis* [53].

## 5. Conclusions

The integration of several types of omics data (phenotypic, transcriptomic, metabolomic) may help understanding plant adaptation to drought and to apprehend the complex mechanisms involved. The investigation of GxE at the transcriptome level has the potential to target interesting candidate genes for further functional analyses. Collecting and gathering omics data from different organs, tissues, genotypes and conditions is the key step for omics breeding in the coming years in order to develop climate resilient crops. The results presented here are therefore valuable resources for the tomato community for further studies intended to decipher drought response mechanisms.

## Figures and Tables

**Table 1:** Enriched gene ontology (GO) terms within the differentially expressed genes under WD in fruit and leaf organs.

Regulation	GO Category	Number of DEGs	Number in Gene Space	Ontology	Corrected *p*-Value	Description
**(A) Fruit**						
down	GO:0003677	62	558	MF	0.0104	DNA-binding
down	GO:0003735	40	172	MF	3.77 × 10^−9^	structural constituent of ribosome
down	GO:0005509	24	134	MF	0.0043	calcium ion binding
down	GO:0005515	210	2233	MF	0.0099	protein binding
up	GO:0008152	71	609	BP	0.0441	metabolic process
up	GO:0016168	13	20	MF	2.61 × 10^−10^	chlorophyll binding
**(B) Leaf**						
down	GO:0003735	117	172	MF	2.98 × 10^−42^	structural constituent of ribosome
down	GO:0007018	25	45	BP	0.0026	microtubule-based movement
down	GO:0008017	19	32	MF	0.0079	microtubule binding
down	GO:0008574	6	6	MF	0.0462	ATP-dependent microtubule motor activity, plus-end directed
down	GO:0009922	15	26	MF	0.0018	fatty acid elongase activity
down	GO:0032183	21	32	MF	1.91 × 10^−5^	SUMO binding
down	GO:0042802	85	245	MF	0.0020	identical protein binding
down	GO:0051082	26	55	MF	0.0030	unfolded protein binding
up	GO:0003700	183	725	MF	0.0028	DNA-binding transcription factor activity
up	GO:0004364	20	52	MF	0.0289	glutathione transferase activity
up	GO:0006468	128	430	BP	3.74 × 10^−5^	protein phosphorylation
up	GO:0008152	161	609	BP	0.0022	metabolic process
up	GO:0045454	27	75	BP	0.0178	cell redox homeostasis
up-down	GO:0003735	23	172	MF	0.0190	structural constituent of ribosome
up-down	GO:0004397	4	5	MF	0.0192	histidine ammonia lyase activity
up-down	GO:0016168	8	20	MF	0.0030	chlorophyll binding
up-down	GO:0031683	5	8	MF	0.0066	G-protein β/γ-subunit complex binding
up-down	GO:0045548	4	6	MF	0.0428	phenylalanine ammonia lyase activity

## Supplementary Materials

The following are available online at https://www.mdpi.com/2073-4425/11/8/900/s1: Figure S1: Phenotypic variation under WD for each genotype. Figure S2: Expression pattern of the consistent DEGs in fruit and leaves. Figure S3: Venn diagram representing the number of genes whose expression level was significantly affected by the genotype, the watering condition and the genotype × watering condition interaction factors in the ANOVA analysis, in fruits and leaves. Table S1: Results of the two-way interactive ANOVA analysis on the phenotypic traits. Table S2 and Table S3: Differentially expressed genes under WD for each of the eight genotypes in fruit and leaves. Table S4: Candidate genes under the WD-responsive QTLs.

## Data Accessibility

The sequences are available at the NCBI SRA repository under project number PRJNA644865. Expression data for both conditions are also available in the TomExpress database http://tomexpress.toulouse.inra.fr.
