# Supplementary material for: Integration of QTL, Transcriptome and Polymorphism Studies Reveals Candidate Genes for Water Stress Response in Tomato
Source: Genes (Basel). 2020 Aug 7;11(8):900. doi: 10.3390/genes11080900 (PMC7465520; doi:10.3390/genes11080900)
Supplement: Supplementary file 1 [file genes-11-00900-s001.zip › Diouf_supplementary/Supplemental Figure 1_4.docx]

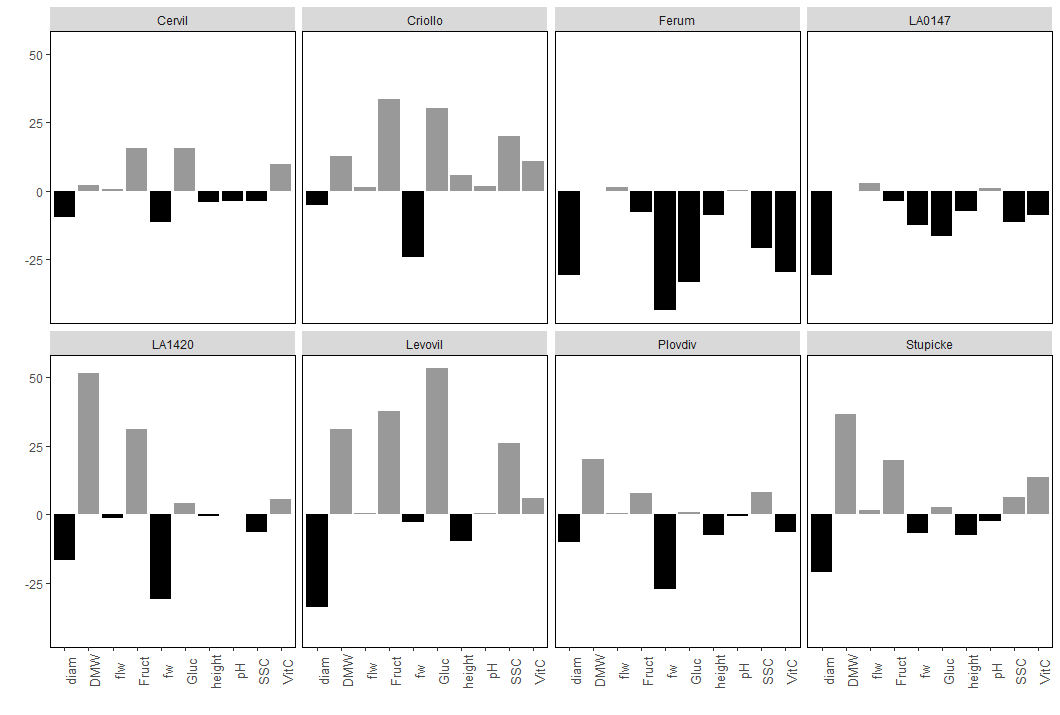


Supplemental Figure 1: Phenotypic variation under WD for each genotype. Phenotypic traits are represented on the x-axis

while the y-axis represents the percentage of increase/decrease of the trait value under the WD condition. In grey and black

are all traits affected positively and negatively by WD condition, respectively.


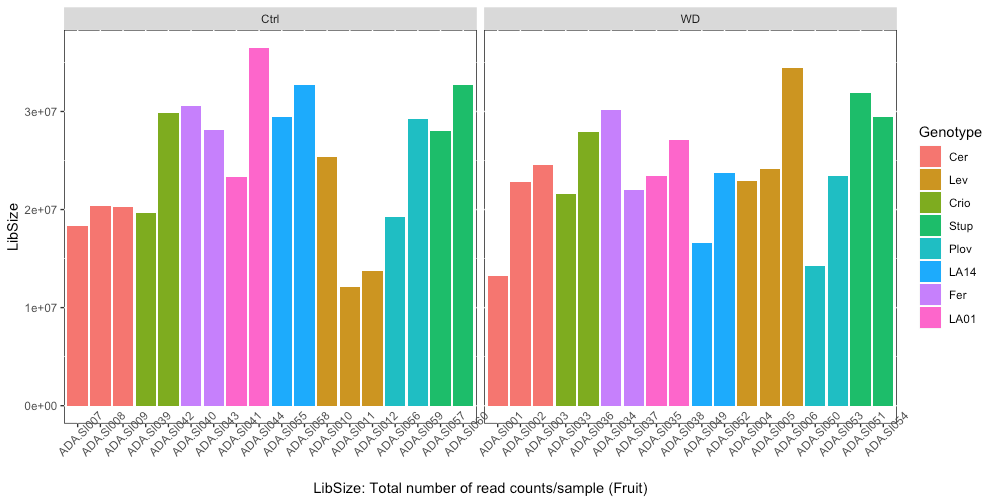


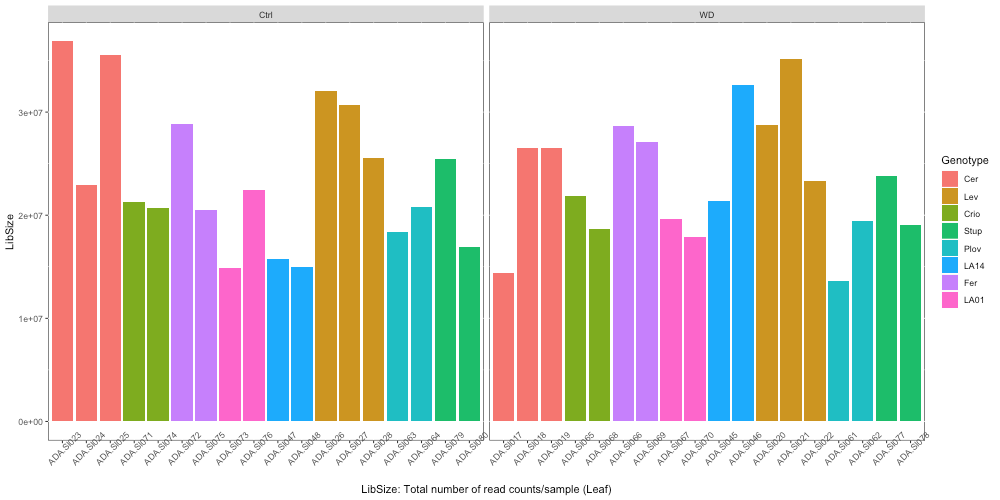


Supplemental Figure 2 A): Total number of read counts after RNA-sequencing processing for fruit. B): Total number of read counts after RNA-sequencing processing for leaf.


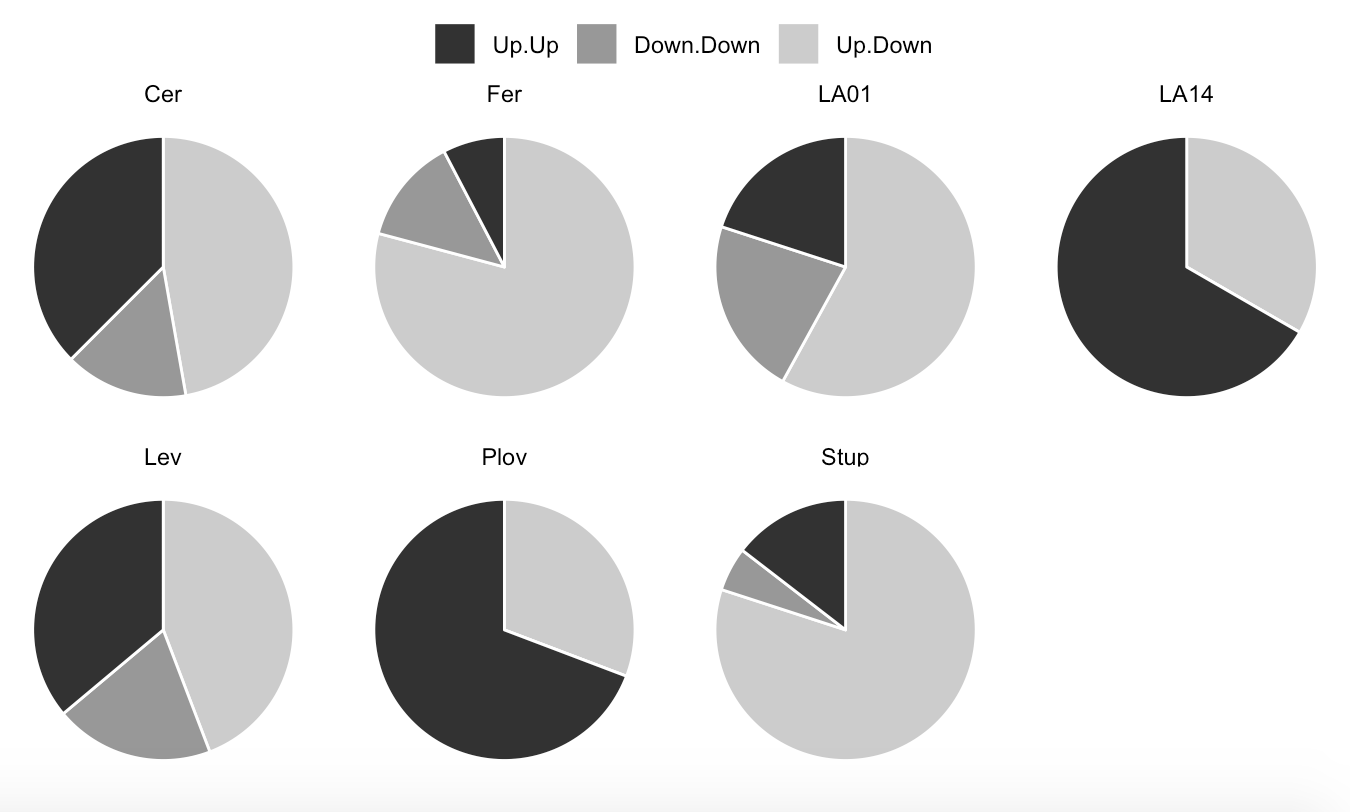


Supplemental Figure 3: Expression pattern of the consistent DEG in fruit and leaf. The DEG were classified as up-up when upregulated in both organs, up-down when up regulated in one organ (leaf or fruit) while down-regulated in the other and

down-down when down regulated in both organs.


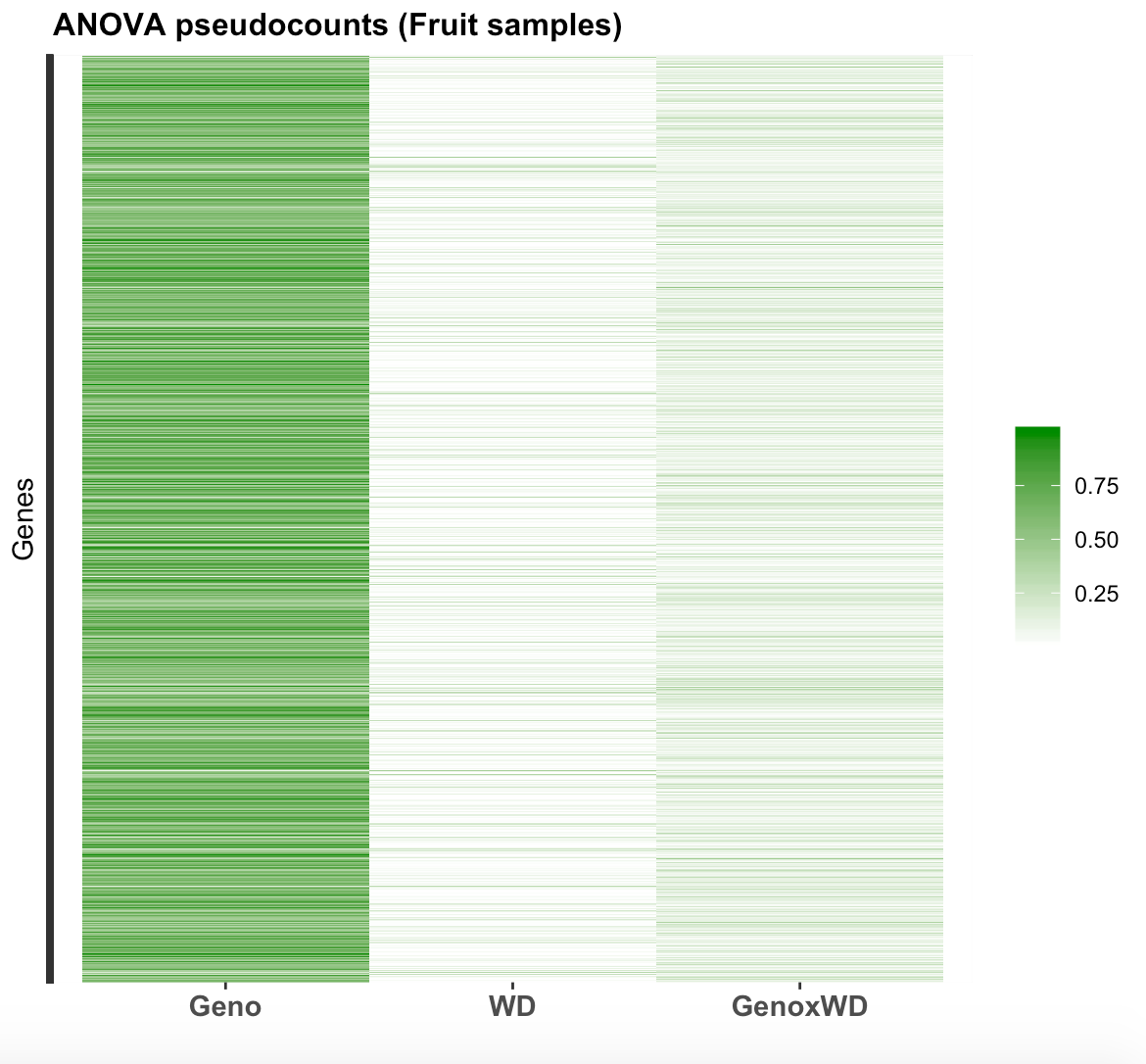


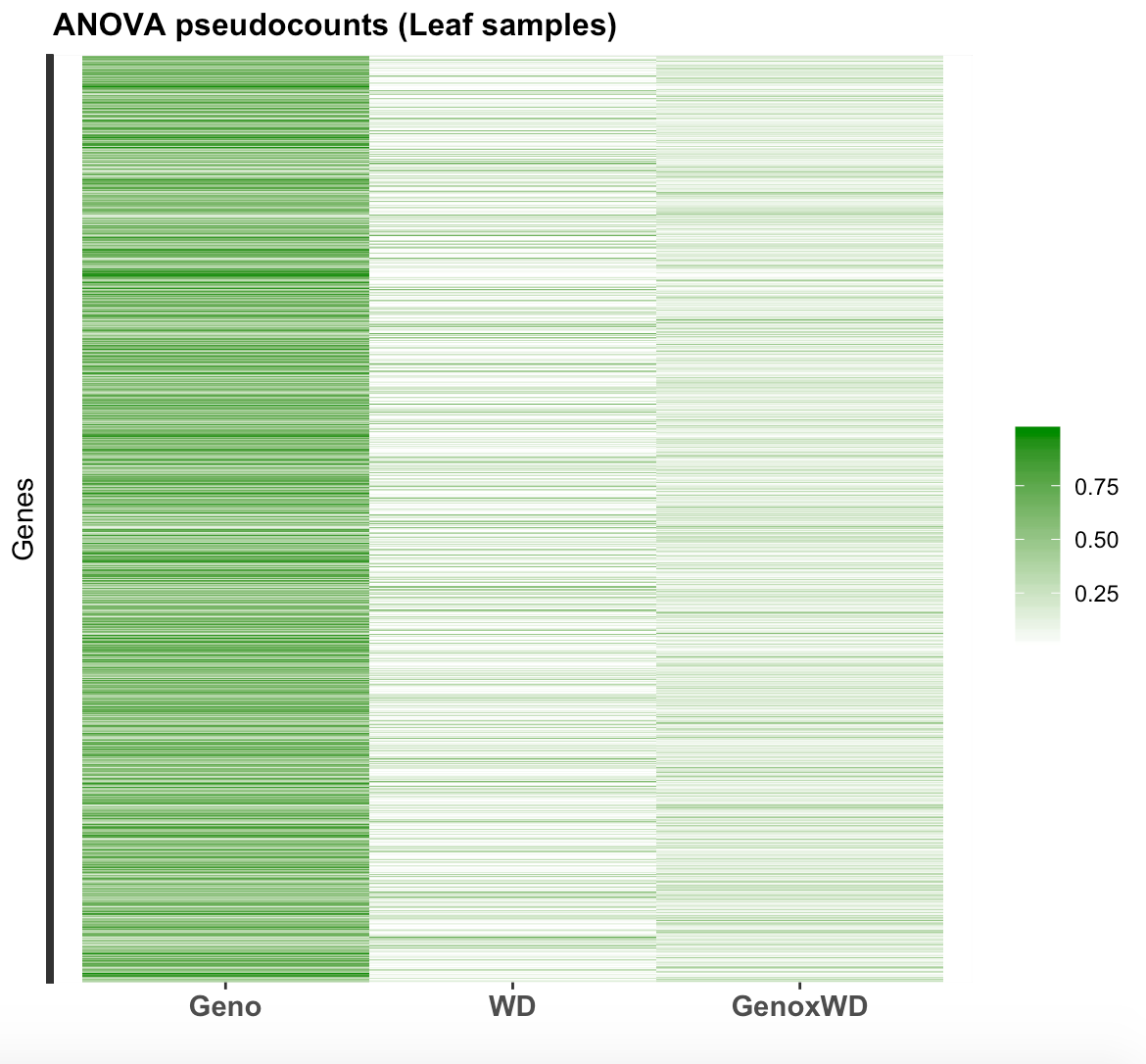


Supplemental Figure 4: Proportion of the sum of square attributed to each factor (Genotype, Condition or GxC) in fruit and

leaf samples through the ANOVA analysis on the normalized transcript level.
