# Supplementary material for: Integration of QTL, Transcriptome and Polymorphism Studies Reveals Candidate Genes for Water Stress Response in Tomato
Source: Genes (Basel). 2020 Aug 7;11(8):900. doi: 10.3390/genes11080900 (PMC7465520; doi:10.3390/genes11080900)
Supplement: Supplementary file 1 [file genes-11-00900-s001.zip › Diouf_supplementary/Table S1.docx]

**Table S1**: Results of the two-way interactive ANOVA analysis on the phenotypic traits. For each factor the associated sum of square and p-value of the test are highlighted. The ns p-values for the GxE factor stand for non-significant GxE.

| **Trait** | **Factor** | **SSQ** | **p-value** |
| --- | --- | --- | --- |
| diameter | Geno | 182.497536 | 1.29E-16 |
| diameter | Env | 166.796934 | 1.15E-19 |
| diameter | GxE | 50.1595955 | 2.22E-05 |
| DMW | Geno | 161.146561 | 4.56E-09 |
| DMW | Env | 16.0913547 | 0.0014867 |
| DMW | GxE | 31.592869 | *ns* |
| flw | Geno | 2701.3381 | 1.08E-55 |
| flw | Env | 1.42074058 | 0.39463091 |
| flw | GxE | 194.378776 | *ns* |
| Fructose | Geno | 12.9844224 | 5.78E-12 |
| Fructose | Env | 1.09974856 | 0.00077117 |
| Fructose | GxE | 3.31885506 | *ns* |
| fw | Geno | 65400.9879 | 1.46E-22 |
| fw | Env | 2519.56424 | 1.22E-07 |
| fw | GxE | 3296.64439 | 6.96E-06 |
| Glucose | Geno | 16.0933101 | 3.60E-16 |
| Glucose | Env | 0.09180106 | 0.15403216 |
| Glucose | GxE | 2.12293183 | 3.75E-05 |
| height | Geno | 35211.5739 | 1.61E-42 |
| height | Env | 1549.16667 | 3.04E-07 |
| height | GxE | 1038.54167 | 0.00876656 |
| pH | Geno | 0.53180262 | 3.90E-06 |
| pH | Env | 0.00832775 | 0.34551452 |
| pH | GxE | 0.34683558 | *ns* |
| SSC | Geno | 100.780933 | 4.75E-13 |
| SSC | Env | 4.67E-05 | 0.99259824 |
| SSC | GxE | 20.87862 | *ns* |
| vitamin-C | Geno | 651.71389 | 0.0001262 |
| vitamin-C | Env | 1.28027436 | 0.77898539 |
| vitamin-C | GxE | 608.99548 | *ns* |
